# Mediastinal lymphangioleiomyomatosis: a case report and literature review

**DOI:** 10.3389/fonc.2025.1588165

**Published:** 2025-09-09

**Authors:** Yike Wang, Quanyong Wang, Lin Shi, Lei Zhao, Yana Dou, Fene Hao

**Affiliations:** ^1^ Department of Radiology, Affiliated Hospital of Inner Mongolia Medical University, Huhhot, Inner Mongolia, China; ^2^ Department of Radiology, Inner Mongolia Autonomous Region Hospital of Traditional Chinese Medicine, Huhhot, Inner Mongolia, China; ^3^ Department of Pathology, Inner Mongolia Medical University, Huhhot, Inner Mongolia, China; ^4^ Computed Tomography System Division, Siemens Medical Systems Ltd, Beijing, China

**Keywords:** lymphangioleiomyomatosis (LAM), mediastinum, extrapulmonary, computed tomography (CT), mediastinal imaging, differential diagnosis

## Abstract

Lymphangioleiomyomatosis (LAM) is a rare disorder that primarily affects women of childbearing age. It is characterized by the abnormal growth of smooth muscle-like cells. While LAM typically occurs in the lungs, it can also be found in the retroperitoneum and pelvis. However, cases originating in the mediastinum are extremely rare. This report discusses an unusual case of mediastinal LAM in a male patient with no abnormal clinical symptoms. The patient, a 70-year-old man, the chest computed tomography (CT) scan revealed an irregular hypodense mass in the left side of the anterior superior mediastinum. Interestingly, the mass did not exhibit significant enhancement in the arterial phase. Instead, it showed striated enhancement in the central area during the venous phase, with no abnormalities observed in the marginal area. To further understand this condition, we conducted a comprehensive review of relevant literature, focusing on the imaging characteristics of mediastinal LAM and the pathogenesis and therapeutic prognosis of LAM. By sharing this information, we aim to enhance understanding and knowledge of this disease.

## Introduction

Lymphangioleiomyomatosis (LAM) is a rare systemic disease classified under the family of PEComas, characterized by the abnormal proliferation of smooth muscle-like cells and cystic lesions (1, 2). The most common site of LAM is the lung, accounting for about 90% of all cases. Extrapulmonary LAM is a rare disease that often occurs concurrently with pulmonary LAM. The primary sites include the pancreas, retroperitoneum, and pelvis, with mediastinal LAM being much rarer (3). LAM primarily affects women of childbearing age, with very few reported cases in males (4). The prevalence of LAM is extremely low, in women, with only 28.7 cases per 1 million persons reported in the literature. While in men, the prevalence of LAM is even lower, at 0.8 per 1 million persons (5). In this study, we conducted a literature review of case reports published in indexed journals from January 1998 to July 2023, focusing on mediastinal LAM. The search was performed using keywords “mediastinal lymphangioleiomyomatosis” in PubMed and CNKI databases. Only five cases of mediastinal LAM were identified in the literature, primarily discussing the pathological manifestations. In this report, we present a case of mediastinal LAM in an elderly man, describe the findings from chest computed tomography (CT), and provide a review of the relevant literature.

To the best of our knowledge, this case study presents the first case of a primary solitary extrapulmonary form of LAM in the mediastinum occurring in a male. We present the following case in accordance with the CARE reporting checklist.

## Case report

**Figure 1:**
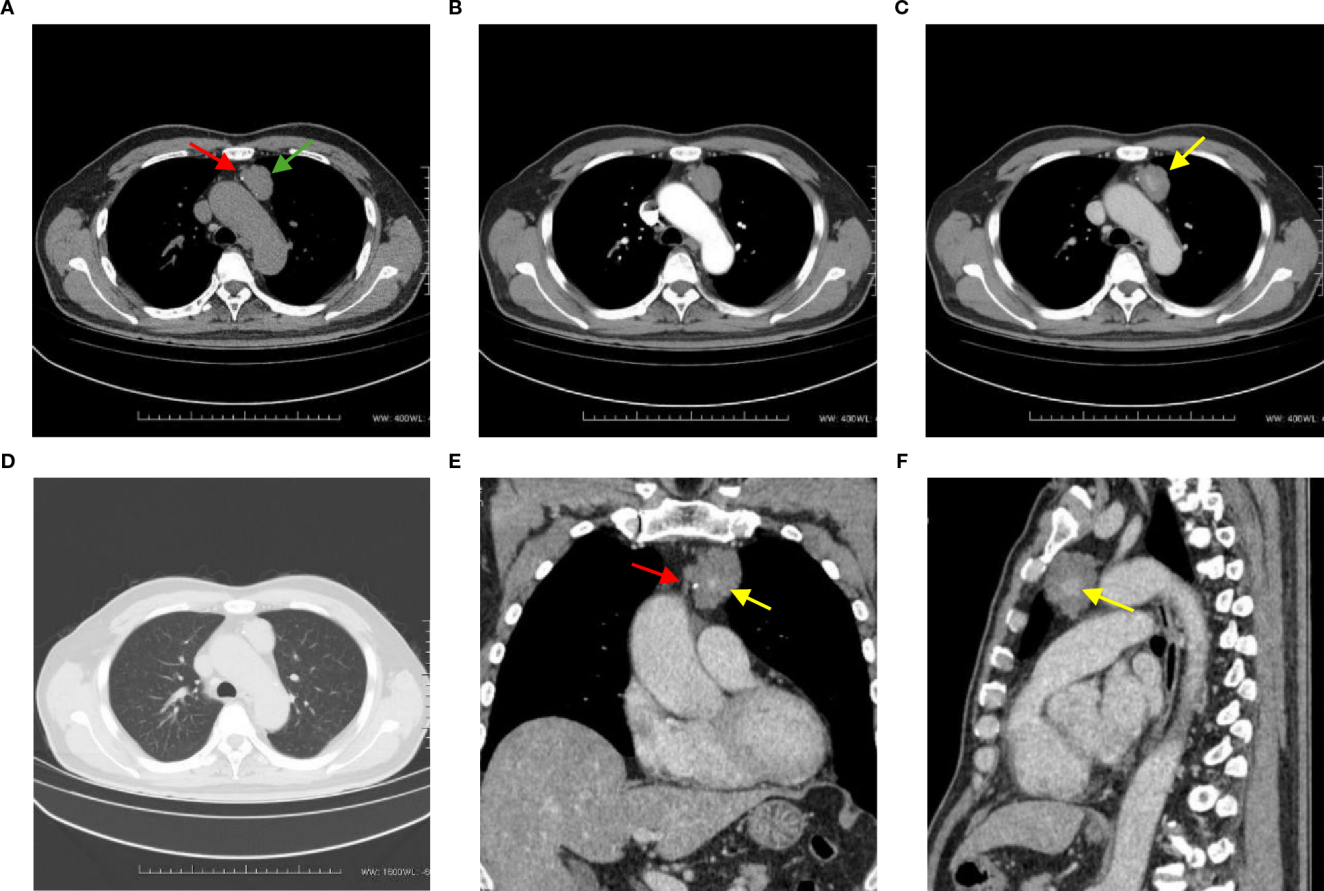
**(A)** This axial CT image shows an irregular hypodense mass in the left side of the anterior superior mediastinum, with clear boundaries (green arrow). Punctate calcification is observed at the edge of the lesion (red arrow). **(B)** This CT image in the arterial phase demonstrates that the mass does not exhibit significant enhancement, with a CT value of 46.1 HU. **(C)** In the venous phase CT image, striated enhancement is observed in the central area of the mass, while no abnormalities are seen in the marginal area (yellow arrow). The CT values are 94.6 HU and 46.3 HU, respectively. **(D)** The lung window image shows a clear texture in both lungs, with no evidence of cystic changes. **(E, F)** Coronal and sagittal reformations further illustrate the striated enhancement in the central area of the mass (yellow arrow), as well as the presence of punctate calcification at the edge of the lesion (red arrow). The mass does not exert any obvious pressure on the surrounding tissues and blood vessels, and there is no evidence of infiltration.

A 72-year-old man presented with an anterior mediastinal mass eight years ago and was recently admitted to the hospital due to an increase in the size of the mass. The patient did not exhibit any abnormal clinical manifestations, family history, medical history, physical examination findings, or abnormal laboratory test results. He had no clinical features suggestive of tuberous sclerosis complex (TSC). Specifically, he did not have central nervous, hydrocephalus, mental retardation. He did not have dermatologic manifestations. He did not have a family member who was diagnosed with TSC. A plain chest computed tomography (CT) scan revealed an irregular hypodense mass measuring 3.5 cm × 3.0 cm × 1.9 cm on the left side of the anterior superior mediastinum. The mass had a CT value of 45.3 HU and displayed clear borders. Punctate calcification was observed at the edge of the lesion (**Figure 1A**). In the arterial phase, the mass did not exhibit significant enhancement, with a CT value of 46.1 HU (**Figure 1B**). However, during the venous phase, the mass showed striated enhancement in the central area, while the marginal area displayed no abnormalities. The CT values in the venous phase were 94.6 HU in the central area and 46.3 HU in the marginal area (**Figure 1C**). No enlarged lymph nodes were observed in the hilum, and the mass did not exert pressure on surrounding tissues or blood vessels. There was also no evidence of infiltration, and the lung window displayed clear lung texture without cystic changes (**Figure 1D**).

Based on a preoperative diagnosis of thymoma, the patient underwent thoracoscopic extended thymic body resection and thoracoscopic thoracic adhesion release. During the surgery, a cystic tumor measuring 3.5 cm × 3.0 cm × 2.0 cm was found in the upper left region of the thymus. The tumor had a regular shape with intact borders. The mediastinal mass, including the thymus gland and bilateral anterior mediastinal fat, was resected while preserving all vital structures.

**Figure 2:**
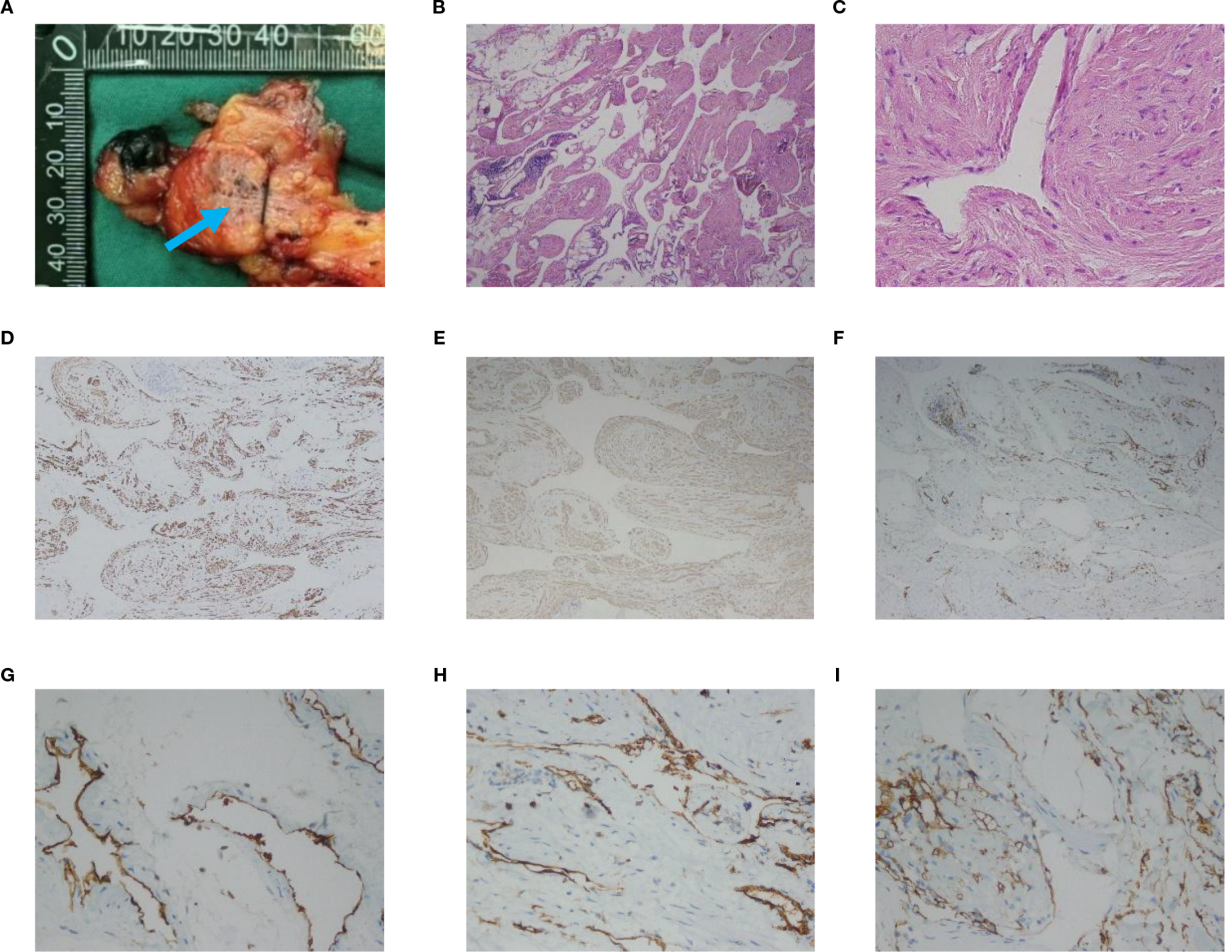
**(A)** Macroscopic examination of the resected specimen reveals clusters of thickened tubular tubules in the local region of the incision (blue arrow). **(B, C)** Histological image shows an endothelial-lined sinusoid cavity surrounded by papillary proliferative spindle-shaped smooth muscle-like cells. HE; **(B)** is original magnification×40; **(C)** is 400× magnification. **(D)** Immunohistochemical staining shows positive staining for Desmin (magnification ×100). **(E)** Immunohistochemical staining shows positive staining for SMA (magnification ×100). **(F)** Immunohistochemical staining shows positive staining for CD31 (magnification ×100). **(G, H)** Immunohistochemical staining shows positive staining for CD31 (magnification ×400). **(I)** Immunohistochemical staining shows positive staining for CD34 (magnification ×400).

Macroscopic examination of the resected specimen revealed a grayish-pink colored mass with clusters of thickened tubular structures in the local region of the incision (**Figure 2A**, blue arrow). Histological examination showed an endothelial-lined sinusoid cavity surrounded by papillary proliferative spindle-shaped smooth muscle-like cells (**Figure 2B**). Immunohistochemical analysis revealed positive staining for Desmin (**Figures 2C, D**), SMA (**Figure 2E**), CD31 (**Figures 2F, G, H**), and CD34 (**Figure 2I**), while staining for D2-40, HMB-45, and MelanA was negative. After correlating the chest CT findings, with these pathological and immunohistological findings, the tumor was diagnosed as mediastinal lymphangioleiomyomatosis (LAM).

The patient had an uncomplicated postoperative recovery and was discharged after seven days. As of one year post-surgery, the patient remains in good health without any pulmonary issues or recurrences. Unfortunately, long-term follow-up imaging beyond one year is lacking.

## Discussion

LAM is a slowly progressive, low-grade, metastasizing neoplasm of women, characterized by infiltration of the lung parenchyma with abnormal smooth muscle-like cells, resulting in cystic lung destruction (1). LAM can also occur outside the lungs, although this is even rarer, with the retroperitoneum and pelvis being the most common sites of occurrence (6–8). The symptoms of LAM are non-specific, with progressive dyspnea, recurrent pneumothoraxes, and chylous effusions being common manifestations in the lungs (9). In the abdominopelvic cavity, LAM typically presents as a painless mass or abdominal distension (10). Due to the rare occurrence of extrapulmonary LAM and its atypical location, extrapulmonary LAM is often difficult to diagnose prior to surgery.

Histopathology is an important criterion of diagnosing LAM. LAM cells are spindle shaped or epithelioid with few mitoses and a bland appearance. These cells have features of smooth muscle that can be identified by antibodies, including HMB-45 antibody (11). LAM tumor cells express estrogen and progesterone receptors. Desmin is myogenic markers for smooth muscle-like cells, while D2–40 and CD34 are marker of lymphatic endothelial cells (12, 13). Immunostaining for HMB-45 is diagnostically useful, although in rare cases LAM lesions do not express this marker (14, 15). Possible causes are technical and biological factors. The test can be combined with other indicators (SMA, ER/PR). Besides, The American Thoracic Society and Japanese Respiratory Society clinical practice guidelines recommend VEGF-D ≥ 800 pg/ml as the threshold for diagnosing LAM (16).

LAM is a multisystem genetic disease caused by germline mutations in TSC genes, TSC1 or TSC2 (17). It occurs in two forms: a sporadic form and a TSC form. The former is a somatic mutation in the TSC gene, and the latter is a germ cell mutation. Statistically, approximately 80% of patients with LAM have a germ cell mutation in either TSC1 or TSC2, and only 15-20% have a disseminated mutation in the TSC gene (18). However, this result is reversed, more than 80% of patients with LAM in pulmonary clinics, registries, and trials have sporadic mutations (19). The reason for this may be the difference in severity between sporadic LAM and TSC LAM, as well as insufficient attention to the pulmonary manifestations of patients with TSC. It has been reported that LAM can be a substantial cause of mortality in patients with TSC. There is a need to intensify genetic analysis to identify TSC mutations in patients with LAM in the future.

LAM has been considered a female-specific disease. However, there are a few LAM cases reported in males. Based on a review of the literature on male LAM cases. There was no significant difference in clinical presentation between TSC-LAM patients and sporadic LAM. TSC-LAM patients were more likely to have renal Angiomyolipoma (AML) and thin-walled pneumothorax in both lungs. One article reviewed the literature of all published male LAM cases from April 1986 to October 2021 and statistically showed that the positivity rates of HMB-45, SMA, Desmin, and estrogen receptor in immunohistochemistry were approximately 40%, 76%, 89%, and 16%, respectively (20).

We report a case of male LAM occurring in the mediastinum. Immunohistochemical analysis revealed positive staining for Desmin, SMA, while staining for HMB-45 was negative. It is suspected that in male LAM, the positivity of SMA and Desmin is higher, while the positivity of HMB-45 is relatively low. Our patient did not receive genetic testing, but he had no history of TSC and no family history. The chest CT revealed no abnormalities in both lungs. Therefore, it is more likely that the patient has sporadic LAM.

Through a review of the literature on LAM reported in indexed journals such as PubMed and CNKI, it was found that there were 19 cases of LAM occurring outside the lungs. Among these cases, 13 occurred in the abdominopelvic cavity, 5 in the mediastinum, and 1 in the head. We summarized five cases of mediastinal LAM (**Table 1**) (6, 21–24).

**Table 1:** 5 cases of mediastinal LAM.

Case report	Age & sex	Location	Imaging Findings	Treatment	Prognosis
Derweduwen et al. (6)	32-year-old woman	upper anterior mediastinum	CT: cystic-solid mass and clear borders	thoracoscopic resection	metastasis to the neck after 2 years
Kataria et al. (21)	23-month-old boy	superior mediastinum	CT: fluid-containing multiloculated cystic mass	surgical excision	survived
Ota et al. (22)	70-year-old woman	anterior mediastinum	CT: round, homogeneous mass with low attenuation, regular margin; MRI: isosignal intensity on T1, high signal intensity on T2, and multiloculated heterogeneous intensity on Gd-enhanced T1-weighted images	surgical excision	survived
Raghuprakash et al. (23)	7-year-old girl	superior mediastinum	MRI: heterogenous T2 hyperintense multicystic lesion	surgical excision	survived
Che et al. (24)	32-year-old woman	Inferior mediastinum	CT: multiple fat density areas	sirolimus	survived

Although there have been studies focusing on the pathological manifestations of mediastinal LAM, the imaging features have not been well-described. Based on a review of the literature on LAM, the imaging features of extrapulmonary LAM can be summarized as follows:

1. The diameter of the mass can vary from 1 cm to 20 cm, and larger lesions (diameter > 3 cm) tend to exhibit obvious cystic changes and may contain chylothorax.
2. CT imaging typically shows a cystic or cystic-solid mass with an irregular or lobulated shape and clear borders. A few reports state that there may be segregation within the mass of abdominal LAM, and the wall of the mass may have varying thickness. During contrast-enhanced scans, there is typically no enhancement in the cystic part of the mass, while the solid part may show uneven enhancement.
3. Lymph node involvement can be seen with LAM, and CT scans may reveal solid nodules with uniform density in the peripheral lymph nodes (25).
4. The masses may exert pressure on adjacent tissues and blood vessels but typically does not infiltrate them.

In our present case, the CT scan showed an irregular hypodense mass with clear borders and punctate calcification at the edge. This is consistent with previous findings reported in the literature (19). The mass did not demonstrate significant enhancement in the arterial phase, while in the venous phase, it showed striated enhancement in the central area and no abnormalities in the marginal area. The central enhancement in the venous phase may be associated with clusters of thickened tubular structures in the center of the mass. When LAM lesions contain solid components, the venous phase of CT may demonstrate mild to moderate enhancement. Pathologically, this is related to the presence of proliferating smooth muscle cells, fibrous tissue, and possible neovascularization within the solid components. The neovascularization may allow the solid components to absorb contrast agents during the CT venous phase, thereby exhibiting enhancement. In this case, the mass did not exert any obvious pressure on the surrounding tissues or blood vessels and did not manifest infiltration. The higher CT value observed on the plain chest CT in this case may be attributed to the high protein content within the lesion.

Mediastinal LAM needs to be differentiated from other mediastinal masses, including thymoma, teratoma, cystic lymphangioma, and lymphoma.

Thymoma: Thymomas are more common in individuals aged 30 to 50, with a higher incidence in men. They are typically located in the anterior and middle superior mediastinum. On CT scans, thymomas appear as irregular masses with uniform soft tissue density (26). Larger tumors may exhibit liquefaction necrosis or calcification (27). Contrast-enhanced CT shows inhomogeneous enhancement. Thymomas often locally invade the pericardium and pleura (28).

Teratoma: Teratomas are more prevalent in females aged 20 to 35 or in their early years. They are usually located in the anterior mediastinum. On CT scans, teratomas present as well-demarcated round unilocular or multilocular cystic lesions with an irregular wall of varying thickness. Punctate calcifications may be present inside the tumor (29). The density of the tumor is uneven, and it may contain calcification, ossification, or fat. Contrast-enhanced CT shows contrast enhancement of the wall and solid components.

Cystic lymphangioma: Cystic lymphangiomas are more common in males and can occur at any age (30). They are typically located in the anterior or middle superior mediastinum. On CT scans, these lesions appear as round multilocular cystic masses with sharp demarcation (31). They usually have a homogeneous water-like density and rarely show calcification (32). On contrast-enhanced CT, the cystic wall and septum show moderate enhancement (33). These tumors are characterized by spreading along the vascular space.

Lymphoma: Lymphomas primarily occur in young adults, with an average age of 30 years. They are mostly located in the anterior mediastinum and commonly present as primary mediastinal diffuse large B-cell lymphomas. On CT, lymphomas appear as irregular or lobulated masses. Larger tumors may contain hypodense necrosis in the center (34). Contrast-enhanced CT shows mild-to-moderate enhancement. Lymphomas often invade adjacent mediastinal structures, such as blood vessels, pleura, and lungs. Pleural and pericardial effusions can be observed in approximately half of the cases (35).

Currently, the rarity of patients with extrapulmonary LAM limits therapeutic measures. Considering the limited understanding of extrapulmonary LAM disease, traditional surgical resection is generally preferred when imaging shows an equivocal diagnosis, However, in patients with milder symptoms, surgical resection may be unacceptable and unnecessary. mTOR inhibitors, such as Sirolimus and Everolimus, are the primary clinical treatment for LAM (36). The American Thoracic Society/Japan Respiratory Society (ATS/JRS) guidelines state that sirolimus improves lung function and quality of life, and it is recommended for patients with LAM who have abnormal or decreased lung function (16). However, sirolimus does not reverse cystic changes or eliminate LAM tumors and may have potential side effects including nausea, diarrhea, mucositis, acne, lower extremity swelling, and hyperlipidemia (37, 38). The combination of surgery and sirolimus may be a more effective strategy for slowing the progression of LAM.

In conclusion, we have reported a rare case of mediastinal LAM with nonspecific clinical presentation and benign histologic characteristics. LAM is a slowly progressive disease that can involve multiple organs simultaneously or sequentially, highlighting the importance of early diagnosis. On CT imaging, mediastinal LAM appears as an irregular hypodense mass with clear borders. During the enhancement scan, the mass does not exhibit significant enhancement in the arterial phase but shows striated enhancement in the central area in the venous phase, with no abnormalities in the marginal area. It should be noted that this conclusion was derived from a single case, and its generalizability is limited. When mediastinal LAM is suspected, radiologists should recommend a chest CT examination. The presence of typical pulmonary LAM imaging features, such as multiple thin-walled cysts of uniform size randomly distributed in both lungs, can be helpful in suggesting the diagnosis of mediastinal LAM. Radiologists should be familiar with the imaging characteristics of mediastinal LAM to consider it as a possibility in the differential diagnosis.

## Data Availability

The original contributions presented in the study are included in the article/Supplementary Material. Further inquiries can be directed to the corresponding author.
